# Clostridioides difficile Infection in the Neurorehabilitation Setting: Importance of a Multidisciplinary Approach and Impact of the Fecal Microbiota Transplantation

**DOI:** 10.7759/cureus.46574

**Published:** 2023-10-06

**Authors:** Daniela Secondo, Dymytrii Massaro, Giulio Verrienti, Francesco Perri, Giuseppe Biscaglia

**Affiliations:** 1 Department of Neurorehabilitation, Casa di Cura Villa Verde, Lecce, ITA; 2 Division of Gastroenterology, Fondazione Istituto di Ricovero e Cura a Carattere Scientifico (IRCCS) Casa Sollievo della Sofferenza, San Giovanni Rotondo, ITA

**Keywords:** fecal microbiota transplantation, neurorehabilitation, role of a multidisciplinary approach, gastrointestinal infections, clostridioides difficile infection

## Abstract

*Clostridioides difficile* infection (CDI) is considered to be one of the most frequent causes of bacterial infectious diarrhea in nosocomial settings. The prolonged hospitalization in bed-ridden conditions and the frequent administration of antibiotic therapy are usually encountered among the risk factors for CDI. Therefore, it is not surprising that CDI rates among rehabilitation hospitals are higher in neurologic facilities. In the neurorehabilitation setting, CDIs, especially if they present with refractory or recurrent aspects, may interrupt the normal course of rehabilitation, influencing, subsequently, the neurological outcomes. CDI treatment depends on the severity of the disease and includes both conservative and surgical approaches, with the latter reserved for severe complicated CDI. Another emerging, highly effective therapeutic option is represented by fecal microbiota transplantation (FMT), which consists of the transfer of screened healthy donor stool to a recipient’s gastrointestinal tract. ​​​In this paper, we report two cases of refractory CDI, affecting patients in the neurorehabilitation pathway; both cases were resolved through FMT. On the one hand, our cases provide more evidence of FMT efficacy in refractory CDIs; on the other hand, they emphasize the need for a multidisciplinary approach to grant the best care to CDI patients.

## Introduction

*Clostridioides difficile* infection (CDI) is one of the most common healthcare-associated infections and a significant cause of morbidity and mortality among hospitalized patients. *Clostridioides difficile* (CD) are Gram-positive, spore-forming, anaerobic bacilli, which are widely distributed in the intestinal tract of humans and animals and in the environment. The transmission of these pathogens occurs in the fecal-oral route. The most important risk factors for CDI include female sex, old age, recent and prolonged hospitalization, bedridden conditions, recent antibiotic therapy, active proton pump inhibitor treatment, active cancer, hypoalbuminemia, and leukocytosis [1]. CD produce two potent toxins: toxin A and toxin B. Toxin A is an enterotoxin with mild cytotoxic activity. It can cause initial damage to the intestinal villi, destroying the brush borders of the membrane. The resulting damage of the intestinal mucosa due to inflammation can lead to its erosion. Meanwhile, toxin B is one of the most potent cytotoxins known, showing a cytotoxic effect 100 times more potent than toxin A. The related main effects include the loss of intracellular potassium and the inhibition of protein and nucleic acid synthesis [2].

The clinical presentation of CDI is blunt and ranges from asymptomatic carrier status, through various degrees of diarrhea, to the life-threatening and sometimes fatal colitis. The diagnosis of CD-induced diarrhea should be suspected in any patient who develops diarrhea within two months of antibiotic use or 72 hours of hospital admission. CDI diagnosis is based on the enzyme immunoassay (EIA) testing for the direct detection of CD toxins or toxin nucleic acid amplification-based assays, both in feces.

Several guidelines on the treatment of CDI have recently been updated. These guidelines identified three CDI severity degrees: non-severe, severe, and severe-complicated (“fulminans”) CDI. Therefore, CDI treatment depends on the severity of the disease and embodies both conservative and surgical approaches [3].

The conservative strategy in CDI management includes the administration of antibiotics and the monoclonal antibody bezlotoxumab (BZT). While vancomycin (VCM) is still proposed by the European Society of Clinical Microbiology and Infectious Diseases (ESCMID) guidelines [4] for the first CDI episode, the Infectious Diseases Society of America and the Society for Healthcare Epidemiology of America (IDSA/SHEA) guidelines [5] suggest fidaxomicin (FDX) preferentially over VCM for initial CDI. This latter is related to the fact that FDX has been demonstrated to be superior in preventing CDI recurrence [3]. 

BZT is a monoclonal antibody against CD toxin B, and its efficacy in preventing recurrent CDI was confirmed in two randomized controlled trials [6]. The IDSA/SHEA guidelines suggest the administration of BZT (along with standard antibiotics) in the case of primary and severe infections [5]. According to the ESCMID guidelines [4], BZT should be given in primary infections only if FDX is not available. Both guidelines agree with BZT use in all patients with recurrent CDIs.

A further and highly effective therapeutic option is fecal microbiota transplantation (FMT), which consists of the transfer of screened healthy donor stool to a recipient’s gastrointestinal tract. Different routes of administration (nasogastric tube, enema, colonoscopy, or capsules) are possible to perform the transfer. In the last 15 years, multiple trials demonstrated that the transplantation of healthy donor feces is an effective therapeutic strategy for recurrent CDI [7]. FMT requires an accurate donor screening; in fact, the potential transmission of viruses and pathogenic bacteria remains possible. A documented transmission of *Escherichia coli* from donors to recipients has induced IDSA/SHEA to restrict FMT to third (or subsequent) CDI recurrence, while the ESCMID and Australasian guidelines [8] suggest FMT for the second recurrence.

Finally, the surgical approach to CDI is represented in most cases by the execution of a total colectomy; the latter should be considered only in the case of severe-complicated (refractory) CDI and reserved to the associated presence of toxic megacolon [3]. 

A study [9] demonstrated that the CDI rates among rehabilitation hospitals are higher in neurologic facilities. Another study [10] revealed that patients admitted to acute neurorehabilitation may have an elevated rate of intestinal colonization with CD without having clinical symptoms. In addition, the prevalence of CD as a source of acute diarrhea in the rehabilitation setting is higher if compared with the prevalence of other microorganisms, such as *Campylobacter*,* Salmonella*,* Shigella*, *Yersinia*, and *Giardia* [11]. As suggested by Arvand [9], rehabilitation facilities should need additional efforts to grant a higher standard in infection prevention and control. In particular, a multidisciplinary approach to the problem (also) in this setting is absolutely essential to achieve the abovementioned goals.

In this article, we present two cases of refractory CDI in the neurorehabilitation setting; the peculiarity of the two cases consists in the fact that CDI interrupted the normal course of rehabilitation of our patients. A coordinated action between different professionals, operating in two separated hospitals - a neurological team and a gastroenterological one - was paramount in treating (and solving) CDI in both cases.

## Case presentation

Case 1

A polytraumatized 17-year-old man was brought to the emergency department (ED) following a motorcycle accident. On admission, the patient presented with a Glasgow Coma Scale (GCS) of 7 (E1-V2-M4). In the emergency room, vital signs included temperature of 36.9 °C, heart rate of 123 beats per minute, and blood pressure of 150/90 mmHg. His past medical history was unremarkable. Because of the neurological status and respiratory distress, the patient underwent orotracheal intubation; soon after, he was moved to computed tomography (CT). Head CT showed in both hemispheres hemorrhagic lesions. A small mesencephalic lesion was also described. In addition, head bone CT demonstrated the presence of multiple fractures of the facial mass (mandible, maxilla, sphenoid sinus walls, major wings of the sphenoid, both orbits, and right carotid canal). Thorax CT reported fractures from the V to X right ribs.

An intracranial pressure sonde was placed and removed after six days because of persistent unremarkable pressure values. The patient was moved to the local intensive care unit (ICU); a percutaneous tracheostomy and a percutaneous endoscopic gastrostomy (PEG) were carried out; a progressive weaning from mechanical ventilation was started. Four weeks later, he was moved to our neurorehabilitation department. On admission, the patient was still deeply sedated. After a progressive suspension of the sedation, it was possible to evaluate his neurological conditions. In particular, the patient presented an impaired consciousness, with the activation of decortication patterns after nociceptive stimulation and inability to follow simple orders.

**Figure 1:**
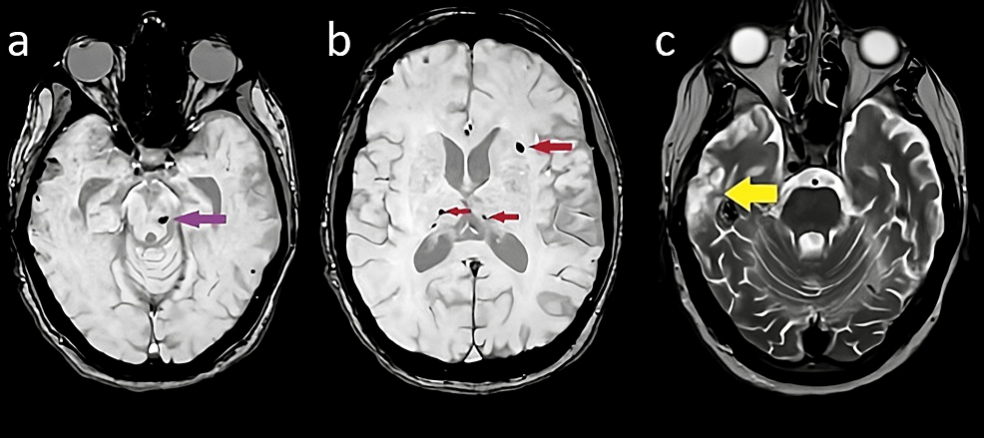
Brain magnetic resonance imaging a, b: Susceptibility weighted imaging (SWI) sequences - showing the presence of multiple hemosiderin deposits (purple and red arrows) - are compatible with a diffuse axonal damage. c: T2-weighted sequence, showing the result of a temporal contusion (yellow arrow) due to the road accident.

To quantify the cerebral, post-traumatic lesions load, we performed a magnetic resonance imaging, which showed the presence of multiple hemosiderin deposits, compatible with diffuse axonal damage (Figure 1).

According to the general health and neurological state, the patient underwent multidisciplinary rehabilitation, which included daily sessions of physiotherapy, speech and language therapy, and neuropsychological rehabilitation. Through these interventions, the clinical and neurological conditions of our patient improved consistently. The patient experienced a good recovery from his consciousness disorder and started to follow simple orders. After an initial phase of relative flaccidity, generalized spasticity developed over few weeks. Because of emerging tonus abnormalities, a therapy with a muscle relaxant (baclofen) was started and administered via percutaneous endoscopic gastrostomy (PEG). A daily dose of 30 mg (10 mg x 3) was initially given; anyway, this dose was progressively increased because of spasticity progression. To achieve the maximum therapeutic benefit, a daily dose of 75 mg of baclofen was ultimately administered. Because only a partial control of the spasticity was reached, we performed a lumbar punction to evaluate the patient’s response to intrathecal baclofen (ITB) therapy. As result of this procedure, the patient was classified as an ITB responder, and he was scheduled for the ITB-pump placement.

Unfortunately, four days before the planned surgical procedure, the patient was found to have fever (38.2 °C) and explosive diarrhea. The laboratory test showed a raised C-reactive protein of 59 mg/dL, white cell count of 12.1×103/µL, and neutrophil count of 8.2×103/µL. A bacteremia could be excluded by several negative blood cultures. In the following days, other cultures (urine and bronchoscopic coltures) resulted negative. From a neurological point of view, no evident changes were detected; in particular, neck stiffness and other meningeal signs were systematically absent. A performed chest X-ray was not indicative for an active infection. We started a symptomatic therapy (including paracetamol and rehydratation) and fever resolved on its own (without administration of antibiotic therapy) within a couple of days. Almost all laboratory inflammatory biomarkers returned to baseline within a few days. What still remained and became persistent was dysentery. In particular, the patient showed more than five bowel evacuations daily.

As multiple risk factors were present, the presence of a CDI was suspected and promptly confirmed by toxin detection in the stool sample. The patient was isolated, and all hygienic precautions were adopted. In the first instance, a VCM therapy (250 mg x 4 via PEG) was started. After the normal course of therapy (10 days), the feces consistency was almost, but not entirely, normal. We decided to extend the VCM cycle to 14 days; unfortunately, in the following days, the patient still presented with diarrhea. An infectious disease consultancy suggested an antibiotic switch-off, replacing VCM with FDX. As recommended, we administered FDX 200 mg every 12 hours via PEG. After 10 days (usual period of therapy), no evident changes in feces consistency were noted. Another antibiotic therapy with tigecycline (TGC), was administered, but this attempt also failed. A combination of FDX with BZT was furtherly attempted, without obtaining a clinical benefit.

During this long period (almost six weeks) of ineffectiveness of the antibiotic therapy, the patient could not profit from a “complete” rehabilitation.

**Figure 2:**
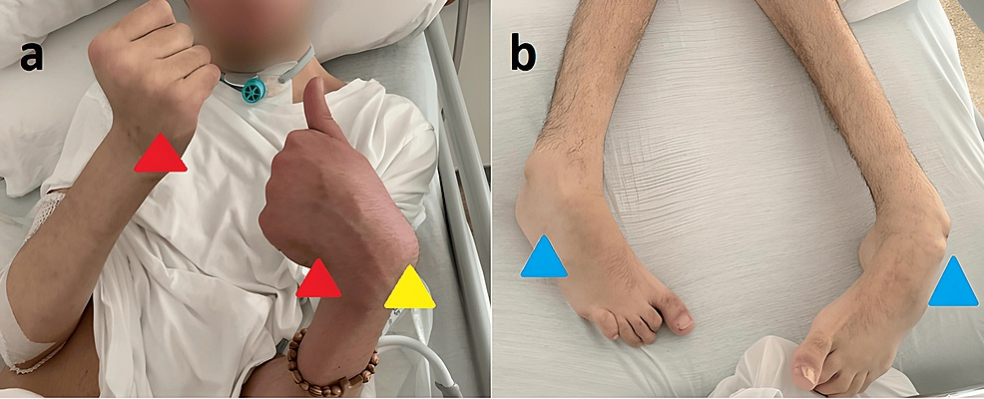
Distal limb deformities due to the spasticity progression Spasticity is a condition in which there is an abnormal increase in muscle tone or stiffness of muscle, which might interfere with movements. The degree of spasticity varies from mild muscle stiffness to severe and uncontrollable muscle spasms. In our case, the spasticity progression - due to the baclofen-dose reduction - led to the development of muscle contractures and distal limb deformities. a: typical aspects of upper limb spasticity with flexed elbows, flexed wrists (yellow arrowhead), and clenched fists (red arrowheads). b: lower limb spasticity affecting both ankles (blue arrowheads)

Even if all the preventive measures to avoid dehydratation and weight loss were adopted, the patient has lost more than four kilograms in six weeks. From a pharmacologic point of view, some drugs (proton pump inhibitors) were interrupted, and the therapy with baclofen was decreased in its dose. In fact, a decrement in the baclofen dose is commonly suggested during a CDI to avoid abnormalities in intestinal motility. As a consequence of this latter, the spasticity led to the formation of distal limb deformities (Figure 2).

In addition, the “isolation status” of our patient did not allow him to profit from the robot-assisted neurorehabilitation.

As the neurorehabilitation process was blocked by the presence of CDI, we contacted the nearest FMT center. In Italy, FMT is (actually) performed only in some selected hospitals. In addition, it is not allowed in underage patients yet [12]. At this time point, our patient has to wait almost two weeks to come of age. In agreement with the parents and FMT center, we waited for this time, and two weeks later, we moved the patient to undergo to FMT. Instillation of feces from an healthy donor into the right colon was performed during a colonoscopy.

To our great surprise, the patient showed no feces abnormalities in the following days. We repeated the direct detection of CD toxins in feces three times in the following weeks, but both toxins were persistently absent. According to the clinical course and laboratory suggestions, the patient was considered recovered from CDI; six weeks later, an ITB pump was placed. At the time of writing of the present article, the patient is still admitted in our rehabilitation department. In the last weeks, the patient has shown a slow but progressive recovery of his neurological deficits. In particular, the spasticity is actually controlled through the ITB therapy. A neuro-orthopedic approach to treat the distal limb deformities has already been planned. 

Case 2

A 64-year-old man was brought to the ED because of the sudden appearance of unresponsiveness and respiratory distress. On admission, the patient was unconscious (GCS: 8). In the emergency room, vital signs included temperature of 37.2 °C, heart rate of 112 beats per minute, and blood pressure of 165/90 mmHg. His past medical history was significant for arterial hypertension and ischemic heart disease. After undergoing orotracheal intubation, the patient was moved to brain CT scan, which showed an ischemic stroke in the left middle cerebral artery (MCA) territory. Angio-CT showed an occlusive disease in the M1 segment of the left MCA and in the left internal carotid due to the presence of thrombus formations. The patient underwent subsequently to endovascular treatment with the placement of two stents in the abovementioned sites, after partial removal of thrombotic clots. Consequently, the patient was admitted in the ICU. Because of a progressive neurological worsening, which was associated to a CT-demonstrated, increased edema in the ischemic territory, the patient underwent to left fronto-parieto-temporal decompressive craniotomy some hours later.

**Figure 3:**
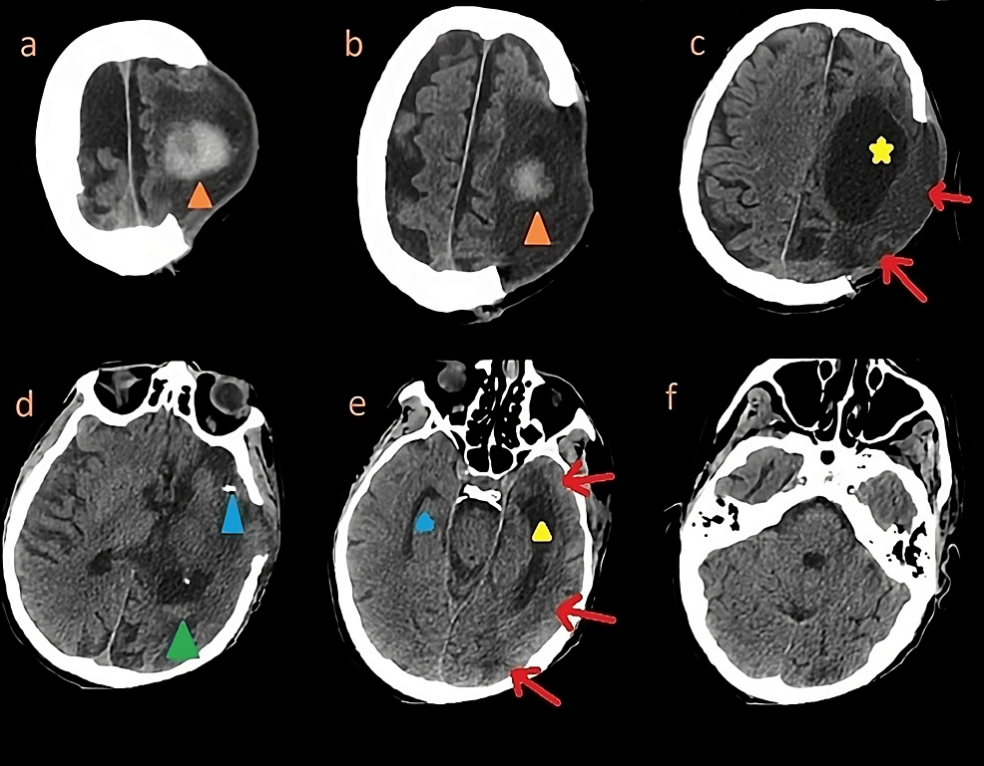
Brain computed tomography (CT) a, b: Organized hematoma in the context of the hemorrhagic transformation of the ischemic stroke (orange arrowheads). c: The lateral ventricle on the left side (yellow star) appears dilated in the context of a hydrocephalus ex vacuo due to the previous territorial stroke (red arrows). d: A stent in the M1 segment of the left middle cerebral artery (blue arrowhead) was previously placed to treat and prevent the occlusive disease; in addition, the presence of a little amount of ventricular blood following the stroke’s hemorrhagic transformation can be demonstrated in the left lateral ventricle (green arrowhead). e: In this section, the presence of a hydrocephalus ex vacuo involving the left ventricle (yellow arrowhead) appears evident. The right lateral ventricle (blue arrowhead) is not dilated. The described hydrocephalus is due to the previous territorial stroke (red arrows). f: No subtentorial lesions were detected.

During the following days, the clinical health and neurological conditions remained critical. A percutaneous tracheostomy and a PEG were carried out; after three weeks, because of a newly diagnosed anisocoria, a brain CT was repeated. This exam showed the hemorragic transformation of the ischemic stroke. After reaching a stable state, a progressive weaning from mechanical ventilation was started. Five weeks later, the patient was moved to our neurologic ICU (n-ICU). On admission, the patient presented awake, with right hemiplegia and global aphasia. We repeated a brain CT, which showed an organized hematoma in the context of the hemorrhagic transformation of the ischemic stroke (Figure 3).

**Figure 4:**
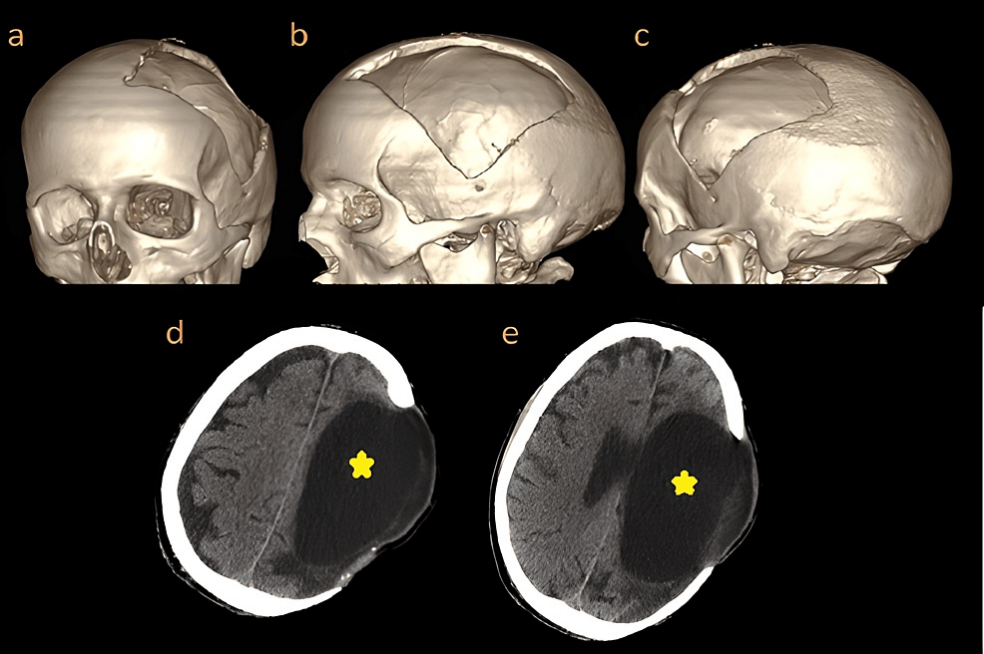
Two- and three-dimensional brain computed tomography a, b, c: The three-dimensional brain imaging was performed to allow the neurosurgical head-bone reconstruction. d, e: Two-dimensional brain computed tomography; the lateral ventricle on the left side (yellow star) appears to be more dilated in comparison with the previous brain imaging session.

The initial clinical course in our n-ICU has been characterized by the onset of multiple infections (involving the airways and urinary tract). Our patient was included in a multidisciplinary early-rehabilitation program, made up of daily sessions of conventional physiotherapy, speech and language therapy, and neuropsychological rehabilitation. In addition, as soon as the resorption process of the hemorragic transformation was completed, we started to verticalize and mobilize the patient with a rehab tilt table (Erigo®, Hocoma, Switzerland) daily. Through this rehabilitative strategy, the patient could improve his neurologic conditions. In particular, after four months from the acute event, the patient's assessment was remarkable for a right hemiparesis (2/5 degree on the Medical Research Council Scale on both the upper and lower limbs). At this time point of the clinical course, the strength improvement was not overall functional; however, considering the initial deficit (hemiplegia without rest-motor faculty) and the brain imaging (compatible with a territory infarct), the reached improvement was rather encouraging. On the other side, no improvements in speech and language were noted. Supposing that the execution of the cranioplasty at this time point could have showed a positive effect on the patient recovery, we performed a three-dimensional brain CT, and we planned a neurosurgical head-bone reconstruction (Figure 4).

Unfortunately, the patient was found to have explosive diarrhea some days before the planned OP. In addition, the patient developed fever (38.0 °C), which resolved with symptomatic therapy. Because a CD was confirmed by toxin detection in the stool sample, we were forced to delay the planned neurosurgical treatment. An antibiotic treatment with VCM (250 mg every six hours) was initially started. In the presence of persistent diarrhea, VCM was replaced by FDX (200 mg twice/day) after five days, according to infectious consultancy. Because of the persistence of high frequency of bowel movements, FDX (after 10 days, i.e., at the end of its cycle) was replaced with TGC. Since this latter attempt also failed, we decided to resort to FMT (according to gastroenterology consultancy), and the patient underwent the same procedure of the previous case.

FMT was performed without complications, and the patient was thereafter moved back to our neurorehabilitation department. Upon return to our clinic and during the following weeks, the patient showed physiologic intestinal movements.

A direct detection of both toxins - done a few weeks later - resulted negative; thus, the patient was finally transferred to the local neurosurgical department, where the planned cranioplasty operation could be finally performed.

## Discussion

In this article, we reported two cases of refractory, non-severe CDIs. In both cases, no criteria for severe or severe-complicated CDI (white blood cell count of >15 000 cells/mL or a rise in serum creatinine level >50% above baseline or core body temperature >38.5°C) were fulfilled. According to the guidelines, we initially administered a conventional antibiotic therapy. The main features of the most used antibiotics in case of CDI are reported in Table 1.

**Table 1:** Main features of the most used antibiotics in the case of Clostridioides difficile infection VCM: vancomycin; FDX: fidaxomicin; MTD: metronidazole; TGC: tigecycline; IDSA/SHEA: Infectious Diseases Society of America/Society for Healthcare Epidemiology of America; ESCMID: European Society of Clinical Microbiology and Infectious Diseases

Antibiotic name	Antibiotic class	Metabolism	Therapy regimen	Indication	Side effects
Vancomycin (VCM)	Glycopeptide	VCM is not metabolized in the liver, and most of the drug is excreted in the urine.	Standard regimen: 125 mg PO 6 hourly for 10 days Tapered/pulsed regime: 125 mg four times daily for 10–14 days, two times daily for 7 days, once daily for 7 days, and then every 2–3 days for 2–8 weeks Higher-dose regimen: 500 mg 6 hourly PO	1. Initial, non-severe episode, as alternative to FDX standard regimen (IDSA/SHEA and ESCMID). 2. First recurrence, non-severe episode: tapered and pulsed regimen OR standard regimen with adjunctive bezlotoxumab (IDSA/SHEA and ESCMID). 3. Severe episode: standard regimen with adjunctive bezlotoxumab if other risk factors for recurrence (e.g., age ≥65 years, immunity compromission) are present (IDSA/SHEA). 4. Severe-complicated "fulminant" episode: higher-dose regimen. In this case, if ileus is associated, an administration of VCM per rectum should be considered (IDSA/SHEA); the ESCMID guidelines suggest the VCM standard regimen and eventual adjunctive TGC.	Common adverse drug reactions (≥1% of patients) associated with oral VCM include abdominal pain, nausea, and dysgeusia (in the case of oral solution).
Fidaxomicin (FDX)	Tiacumicin (new class of narrow spectrum macrocyclic antibiotic)	The drug is metabolized to its active metabolite via hydrolysis of the isobutyryl ester, which occurs primarily in the intestine and therefore does not depend on hepatic CYP450 enzymes.	Standard regimen: 200 mg PO 12 hourly for 10 days Extended-pulsed regimen: 200 mg PO 12 hourly for 5 days followed by 200 mg PO every other day for 20 days	1. Initial episode, non-severe: standard regimen (IDSA/SHEA and ESCMID) 2. First recurrence, non-severe episode: standard regimen (IDSA/SHEA and ESCMID) or extended-pulsed regimen (IDSA/SHEA) 3. Severe episode: standard regimen (IDSA/SHEA and ESCMID) 4. Severe-complicated "fulminant" episode: standard regimen (ESCMID)	The most common side effects include nausea, abdominal pain, vomiting, anemia, neutropenia, and gastrointestinal hemorrhage.
Metronidazole (MTD)	Nitroimidazol	MTD is metabolized in the liver and undergoes biotransformation through hydroxylation, oxidation of side chains, and glucuronidation. Both unaltered MTD and its metabolites are excreted primarily by the kidney, although biliary excretion does occur.	Standard regimen: 500 mg PO 8 hourly for 10–14 days	1. Initial episode, non-severe: standard regimen only if VCM and FDX are not available (IDSA/SHEA and ESCMID)	Common side effects include nausea, a metallic taste, loss of appetite, and headaches.
Tigecycline (TGC)	Tetracycline	A slight amount of metabolism may occur via glucuronidation. TGC is mainly eliminated as unchanged drug and metabolites in the bile and feces (59%). Another 22% of the drug is excreted as unchanged drug in the urine.	Standard regimen: 100 mg for the first intravenous administration (antibiotic load) and then 50 mg 12 hourly	TGC should be considered in the case of severe-complicated, "fulminant" episode (ESCMID)	Gastrointestinal symptoms are the most common reported side effect.

Both cases were unresponsive to the first-line therapy (VCM and FDX) and even to subsequent lines of antibiotic therapy, showing refractory features.

“Refractory CDI” is a CDI that is unresponsive to treatments, leading to persistence of diarrhea (with positive CD toxins or with negative CD toxins but in the absence of other possible causes of diarrhea) [13]. For patients suffering from refractory, non-complicated CDI, the ESCMID guidelines suggest reconsidering the diagnosis, since VCM and FDX resistance is rare. In fact, an alternative diagnosis in combination with CD colonization may be consistent in a significant number of “supposed” refractory CDI patients. According to both guidelines, we excluded other common causes of persistent diarrhea; subsequently, we administered “second-line” antibiotics (TGC), without obtaining significant effects. Finally, both cases were resolved by rescue FMT. In fact, after performing FMT, we noted the immediate diarrhea disappearance; furthermore, the direct detection of CD toxins in feces (eight weeks after FMT) was - in both cases - negative.

Both abovementioned guidelines suggest FMT preferentially in the case of CDI recurrence or severe CDI, while the Australasian guidelines [8] recommend FMT in the case of medical therapy failure.

A peculiarity of the presented cases consists in the fact that our patients were included in the neurorehabilitation pathway. In this context, solving CDI through FMT allowed overcoming a dangerous impasse that was causing a harmful delay in the patient care pathway. The importance of an integrated, multidisciplinary approach in the management of CDI patients in the neurorehabilitation setting should be underlined. In fact, the cooperation between different specialized professionals (neurorehabilitation specialists and specialists from other disciplines, e.g., gastroenterology, internal medicine, infectious diseases medicine, and nutrition service professionals) is absolutely needed in improving treatment efficiency and patient care. Only the integration of all these professionals is effective in granting an optimal treatment of this infection and of its consequences, especially in the neurorehabilitation context. 

A recent study [14] on a small number of chronic stroke patients who experienced CDI during the rehabilitation process demonstrated that these patients do not show statistically significant difference in rehabilitation outcomes when compared to the control group. Even if these results may be consistent in the chronic rehabilitation phase, future research should demonstrate if similar results could be extended to patients in the acute and sub-acute neurorehabilitation setting. Some considerations on this point may be taken into account. As presented in our cases, an effective neurorehabilition may request the execution of different surgical interventions, and these treatments are (obviously) contraindicated in the case of active CDI. In fact, some inpatients in the neurorehabilitation setting may benefit from a number of invasive procedures (i.e., cranioplasty, ITB-pump placement, diagnostic and therapeutic lumbar punctions, and ventriculoperitoneal shunt placement), especially in the acute and sub-acute phases. Even if there are no sufficient data on this topic in the literature, it is reasonable to suppose that a delay of invasive procedure due to the CDI presence may lead to worst neurorehabilitative outcomes.

In addition, the isolation of CDI patients is mandatory. As a consequence, CDI patients in the neurorehabilitation setting are mostly treated by rehabilitative staff in their room, and the entry to appropriate rehabilitation gyms is denied due to infectious risks. Moreover, the continuous stimulation related to the surroundings and the interactions with other patients are limited. For the same reasons, the accessibility to the robot-assisted rehabilitation is restricted in the case of active infections. Future research should also clarify the impact of these limitations on the neurorehabilitation outcome.

## Conclusions

There is a paucity of literature about CD and neurorehabilitation; from a mere neurorehabilitative point of view, delayed treatments bring to poorer neurological outcomes. CDIs may cause a delay of important therapeutical interventions in the acute neurorehabilitation setting, therefore influencing the neurological outcome. In this context, FMT should be considered as an important resource, especially in the case of medical failure. Even if FMT presents some complexities in immunosuppressed patients and requires an accurate donor screening, this procedure seems to be effective and well tolerated also in the acute phase of the neurorehabilitation pathway. Future studies should definitively clear the impact of CDI on outcomes during the acute phase of the neurorehabilitation. Finally, FMT feasibility in acute neurologic patients should be further pursued and investigated.

## References

[1] Czepiel J, Dróżdż M, Pituch H (2019). Clostridium difficile infection: review. Eur J Clin Microbiol Infect Dis.

[2] Mounsey A, Lacy Smith K, Reddy VC, Nickolich S (2020). Clostridioides difficile Infection: update on Management. Am Fam Physician.

[3] Bishop EJ, Tiruvoipati R (2022). Management of Clostridioides difficile infection in adults and challenges in clinical practice: review and comparison of current IDSA/SHEA, ESCMID and ASID guidelines. J Antimicrob Chemother.

[4] van Prehn J, Reigadas E, Vogelzang EH (2021). European Society of Clinical Microbiology and Infectious Diseases: 2021 update on the treatment guidance document for Clostridioides difficile infection in adults. Clin Microbiol Infect.

[5] Johnson S, Lavergne V, Skinner AM, Gonzales-Luna AJ, Garey KW, Kelly CP, Wilcox MH (2021). Clinical practice guideline by the Infectious Diseases Society of America (IDSA) and Society for Healthcare Epidemiology of America (SHEA): 2021 focused update guidelines on management of Clostridioides difficile infection in adults. Clin Infect Dis.

[6] Wilcox MH, Gerding DN, Poxton IR (2017). Bezlotoxumab for prevention of recurrent clostridium difficile infection. N Engl J Med.

[7] Ghani R, Mullish BH, Roberts LA, Davies FJ, Marchesi JR (2022). The potential utility of fecal (or intestinal) microbiota transplantation in controlling infectious diseases. Gut Microbes.

[8] Trubiano JA, Cheng AC, Korman TM (2016). Australasian Society of Infectious Diseases updated guidelines for the management of Clostridium difficile infection in adults and children in Australia and New Zealand. Intern Med J.

[9] Arvand M (2018). Clostridioides difficile in neurological rehabilitation: Prevalence, risk factors and distribution of virulent strains. Neurol Rehabil.

[10] Marciniak C, Chen D, Stein AC, Semik PE (2006). Prevalence of Clostridium difficile colonization at admission to rehabilitation. Arch Phys Med Rehabil.

[11] Yablon SA, Krotenberg R, Fruhmann K (1993). Clostridium difficile-related disease: evaluation and prevalence among inpatients with diarrhea in two freestanding rehabilitation hospitals. Arch Phys Med Rehabil.

[12] (2023). National program on human fecal microbiota transplantation - Ministry of Health [Article in Italian]. http://www.trapianti.salute.gov.it/imgs/C_17_cntPubblicazioni_416_allegato.pdf.

[13] Cammarota G, Ianiro G, Tilg H (2017). European consensus conference on faecal microbiota transplantation in clinical practice. Gut.

[14] Chiaramonte R, D'Amico S, Marletta C, Grasso G, Pirrone S, Bonfiglio M (2023). Impact of Clostridium difficile infection on stroke patients in rehabilitation wards. Infect Dis Now.

